# A Zebra in Horse's Clothing: Rethinking the Diagnosis of Rare Diseases

**DOI:** 10.1002/mgg3.70172

**Published:** 2025-12-22

**Authors:** Rajeev Dutta, Cathy Duong, Virginia Kimonis, Changrui Xiao

**Affiliations:** ^1^ School of Medicine University of California, Irvine Irvine California USA; ^2^ Department of Philosophy University of California, Irvine Irvine California USA; ^3^ Division of Genetics and Metabolism, Department of Pediatrics University of California, Irvine Irvine California USA; ^4^ Department of Neurology University of California, Irvine Irvine California USA; ^5^ Department of Pathology University of California, Irvine Irvine California USA; ^6^ Children's Hospital of Orange County Orange California USA

**Keywords:** diagnosis, genetic testing, rare and undiagnosed diseases, zebra

## Abstract

**Introduction:**

Rare diseases sometimes present with deceptively common symptoms, complicating diagnosis and decisions about genetic testing. While testing for rare disease offers important benefits, it also carries risks that warrant careful consideration.

**Methods:**

We review illustrative cases of rare diseases, along with current screening and diagnostic practices, to reexamine guiding principles for genetic testing. The analysis focuses on balancing clinical utility, patient‐centered care, and broader policy implications.

**Results:**

We propose a number of recommendations to guide testing, including ruling out common causes before proceeding, ensuring the presentation is atypical for other common conditions, confirming consistency with a specific, treatable rare disease entity or group, assessing patient or family capacity for informed decision‐making, and matching test invasiveness to expected diagnostic utility. Comparison with newborn screening and diagnostic testing highlights discrepancies between these principles and common practices, highlighting the difficulty of achieving consistency.

**Conclusions:**

Establishing uniform guidelines for genetic testing remains challenging, particularly given the limited knowledge surrounding rare disorders. Coordinated efforts are needed to protect patient interests, assess the utility of diagnoses across varied contexts, and ensure that both clinical practice and policy development maximize benefits while minimizing harms.

## Graphical abstract

We analyze rare diseases with common presentations to reexamine genetic testing principles. In proposing criteria that emphasize patient capacity, test utility, and proportional invasiveness, we highlight gaps between guidelines and current practice. Coordinated, patient‐centered approaches are critical to maximize benefits, minimize harms, and inform policy in diagnosing rare disorders.

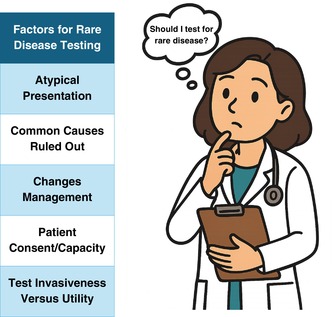

## 1 Introduction

A familiar diagnostic challenge occurs when common diseases have rare presentations—further work‐up, evaluation, and testing are often needed to crack these difficult cases. But the mirror image, in which a rare disease appears deceptively as a common presentation, is not well‐explored. In the first type of challenge, additional investigation must take place before a (correct) diagnosis that satisfies the clinician is found. For example, a patient with appendicitis might present unusually with right upper quadrant pain (rather than the typical right lower quadrant pain) if the patient has an anomalously‐located appendix, resulting to initial, misleading suspicions of hepatobiliary involvement (Hafiz et al. 2017). In the second type of challenge, by contrast, a diagnosis that satisfies the clinician may discourage the additional investigation that would have led to a more precise diagnosis. Moreover, rare diseases may be indistinguishable from common diseases emerge early in the disease course or in relatively mild forms. These factors contribute to people with rare diseases remaining undiagnosed for many years (or indefinitely—see Sirrs et al. 2016; Pérez‐López et al. 2017).

Consider that patients with atypical presentations of lysosomal storage diseases, like juvenile‐onset Tay‐Sachs disease and mucopolysaccharidosis III, may present with primarily psychiatric symptoms (e.g., psychosis, cognitive decline—see MacQueen et al. 1998; Xiao et al. 2022). Such patients may fit criteria for schizophrenia or dementia (including the exclusion of organic causes like thyroid dysfunction, vitamin B_12_ deficiency, etc.) and thus be (mis)diagnosed. Though the *Diagnostic and Statistical Manual of Mental Disorders, 5th edition* criteria require “excluding organic causes,” there are no clear guidelines or consensus on the practical application of this clause (American Psychiatric Association 2022). While problems such as thyroid dysfunction or vitamin B_12_ deficiency are routinely tested in the workup for these disorders, rarer conditions such as neuropsychiatric lupus or NMDA‐receptor encephalitis are less commonly tested (Steiner et al. 2013; Gibson et al. 2019; Tiosano et al. 2017). Genomic sequencing, which is required for the evaluation of many ultra‐rare conditions, is almost never performed. At the same time, however, patients with ultra‐rare conditions with deceptively common presentations are seriously harmed by a lack of diagnosis, like a psychiatric hospitalization that may occur due to the psychosis‐predominant presentation of lysosomal storage diseases (Jina‐Pettersen 2022).

A dilemma follows from these cases: Should patients presenting primarily with psychiatric be tested for lysosomal storage diseases? On the one hand, testing for these diseases in all patients with the deceptively common presentation would provide answers, community, reproductive risk assessment, access to clinical trials, and (perhaps one day) treatment options for the minority of patients, who actually do have rare diseases. That is, widespread testing enables potential for beneficence to patients that have the disease, increases the potential for autonomy (e.g., in selecting treatment options and future family planning), and contributes to distributive justice (i.e., by increasing the accessibility of testing and therapeutics). On the other hand, this widespread use of testing would cause immense distress and unnecessary demand on resources for the majority of patients, who don't have rare diseases and are more likely to receive a false positive result. That is, widespread testing may strongly violate the principles of non‐maleficence, autonomy (e.g., necessitating further testing in response to false positives), and justice (e.g., through healthcare resource waste). Given how rare disorders in these categories are, a straightforwardly utilitarian approach might demand exclusion of such testing from diagnostic guidelines on account of the harm that would result for those without rare diseases. Nevertheless, do we have a duty to provide testing for rare diseases for patients with common presentations? If so, when? If not, why?

This article aims to examine the ethics of including or excluding rare disease testing from the diagnostic workup. In particular, we consider whether (and when) pursuing testing for rare diseases is appropriate, suggesting criteria for inclusion or exclusion that considers the rarity, severity, testability, and treatability of these diseases, including the utility of the diagnosis to the patient. By also considering cases in which rare diseases emerge with deceptively common presentations, we aim to widen the scope of our recommendations to ensure their robustness.

We begin by detailing a number of examples of the unique phenomenon in question—when rare diseases mimic common diseases—to extract generalizable lessons from extant literature on the advisability of testing for rare diseases under various conditions (Section 2). Then, we apply these lessons to outlining ethical considerations for testing for rare diseases potentially mimicking common diseases, guided by the principles of biomedical ethics, particularly distributive justice and non‐maleficence (Section 3). Next, we examine discrepancies between these ethical considerations and real‐life practices (Section 4). Ultimately, we conclude that diagnostic algorithms ought to balance population‐level efficiency and individual‐level equity, though this balance is conceptually and logistically difficult to strike, especially when considering the future challenges in the diagnosis of rare disease (Section 5).

## 2 Rare Diseases and Their Diagnosis

First, we outline a few examples of the phenomenon in which rare diseases display deceptively common presentations before turning to robust recommendations for rare disease testing. Juvenile‐onset Tay‐Sachs Disease, as previously mentioned, can present with psychosis in up to half of all cases, leading to a diagnosis of (usually hebephrenic) schizophrenia (MacQueen et al. 1998).

Mucopolysaccharidosis type III may manifest as cognitive decline, leading to an imprecise diagnosis of ADHD or early‐onset dementia (Xiao et al. 2022; Pará et al. 2021) Adult‐onset Ornithine Transcarbamylase Deficiency may present with hyperammonemic encephalopathy, which may incorrectly incline a clinician toward a diagnosis of hepatic encephalopathy associated with alcohol use disorder (Niclasen et al. 2022). In cases like these, patients receive therapeutics that are standard of care for the common diagnosis but ineffective in the precise diagnosis (e.g., high‐dose clozapine for a patient with Tay‐Sachs disease) and often go undiagnosed for several years. These factors contribute to the so‐called “diagnostic odyssey,” in which patients and their families undergo arduous, costly, and lengthy procedures and clinic visits in hopes of receiving an accurate diagnosis (Bauskis et al. 2022).

The failure to diagnose these patients is evidently harmful, but the question of screening for these disorders raises the key ethical dilemma: Should we regularly screen for rare genetic disorders in the setting of common presentations (e.g., psychosis, cognitive decline, etc.)? The answer to this question will prove helpful for the diagnosis of rare disease more broadly as well. We turn to lessons from existing practices and ethical considerations for genetic testing.

At one end of the spectrum, there is newborn screening for genetic disorders like phenylketonuria, which is mandatory across the United States and strongly recommended across Europe, including in the United Kingdom (Berry et al. 2013; Loeber et al. 2021). These screening guidelines persist for all individuals, although phenylketonuria is particularly rare in certain demographic groups (e.g., people of Asian or South American descent—see Hillert et al. 2020). At the other end of the spectrum, in cases like those mentioned above (e.g., a patient presents with psychosis), genetic testing is never standard of care. Investigating the cases between these ends and understanding the factors that, intentionally or unintentionally, motivate screening in those cases is a natural next step (Kruse et al. 2022). As an example, an emerging trend of screening dialysis patients for Fabry disease has appeared across a number of nations—prevalence values of up to 1.2% in this population have been reported (Frabasil et al. 2019; Kabalan et al. 2013; Kotanko et al. 2004; Nakao et al. 2003; Porsch et al. 2008). This strategy is particularly effective in detecting Fabry disease in a high‐risk population, since its disparate clinical features such as cardiomyopathy, angiokeratomas of the skin, pain in the extremities, strokes, and corneal whorls may not be connected by clinicians to make a unifying diagnosis.

Genetic testing may be limited by the availability of resources in local or national laboratories to test patients (Cox et al. 2003). This may be especially true for ultra‐rare disorders. There may even be disorders for which tests to do not exist, which precludes testing. Examples of these may include hyper‐rare disorders, which affect < 1/10^8^ of individuals (compared to ultra‐rare disorders, which affect < 1/50,000 of individuals) meaning that there may be only one or even no living patients with the disorder (Smith et al. 2022). Another consideration is equivocal test results, like variants of uncertain significance (VUS), which can be misleading, add to uncertainty or stress for patients, and trigger unnecessary surveillance, all without providing the answers they hoped to receive through genetic testing (Clift et al. 2020). Genetic testing without a directed clinical suspicion may be undesirable for clinicians as it increases the likelihood of results with limited clinical utility, such as VUSs. Thus, disorders with affordable, available, and reliable tests may be more likely to receive testing and an accurate diagnosis.

The overall clinical picture may increase the chances of screening for a rare disease. Consider Hutchinson‐Gilford Progeria Syndrome, which has striking dysmorphic features (e.g., disproportionately large head size, narrow nasal ridge, etc.) that may lead a clinician to order testing for the disorder (Gordon et al. 2023). Thus, consistency of the presentation with a recognized clinical entity may increase the likelihood, and effectiveness, of testing. Of course, other considerations can outweigh this one, notably newborn screening for disorders in which the natural history is predictable and significant or for which early intervention is critical for treatment.

Further, the treatability of disorders, if diagnosed, can impact the desirability of screening (Fuentes and Martín‐Arribas 2007). Consider Huntington's Disease, in which the absence of a therapy addressing the lethality the disease may lower the actionability or desirability of genetic testing for a particular patient (Terrenoire 1992). This consideration may be exacerbated as next‐generation sequencing and other advanced modalities for detecting genetic disorders outpace the development of therapeutics for these disorders (Boycott et al. 2013). In such cases, diagnosis may actually be harmful to certain patients. For example, interactions with family members, insurance companies, governmental agencies, employers, etc., may be negatively impacted regardless of legislation in place to dissuade genetic discrimination, in part through stigma associated with genetic disorders, by diagnosis with a harmful, untreatable genetic disorder (Pulst 2000). Yet, this property of treatability cannot be divided neatly into the two categories of treatable and untreatable. There may be interventions that treat symptoms of the disease (e.g., immunosuppression or organ transplant) without addressing the underlying disease. There may be disease‐modifying therapies (e.g., gene therapies). Further, there may be research efforts directed toward treating the disease that are at various stages of development and testing (e.g., Phase I versus Phase III trials). Finally, diagnosis can lead to risk assessment or subsequent disease‐modifying interventions for family members.

There are numerous complex factors that contribute to the desirability of genetic testing, including the timing of testing, regulatory matters, accessibility, consequences of incidental findings, family planning, involvement of relatives, economic interests, privacy concerns, and more (Kruse et al. 2022). With these examples, we proceed to examine some ethical considerations for testing.

## 3 Ethical Considerations for Testing

In assessing ethical considerations for rare disease testing with common presentations (e.g., psychosis, cognitive decline), the principles of biomedical ethics are essential guides. Non‐maleficence, distributive justice, beneficence, and respect for autonomy are each paramount in weighing whether this testing is appropriate (Varkey 2021).

Non‐maleficence is considered primarily with respect to the risk of false positives and VUSs, which are more likely to occur for ultra‐rare diseases than for common diseases (Akle et al. 2015). A brief mathematical example can illustrate the problem. Take 3 million as the number of people meeting criteria for schizophrenia in the United States (McGrath et al. 2008). The prevalence of late‐onset Tay‐Sachs disease is estimated to be around 1 out of everyone 320,000 people in the United States (Lui et al. 2024). Suppose we have a test with a relatively low, 1% false positive rate (a test and number devised hypothetically for the purpose of this thought experiment). If all 3 million people were tested for late‐onset Tay‐Sachs disease were tested, we could expect around 30,000 false positives, compared to around 9 or 10 true positives. In other words, testing everyone who meets criteria for schizophrenia for this ultra‐rare genetic disease would result in 3000 false positives for every true positive. The utilitarian arithmetic seems to stand strongly against testing patients on the grounds of non‐maleficence, given the immense harm to patients in receiving false positive results (White and Algeri 2023). Moreover, this ethical harm is not merely from a failure to appropriately implement or conduct testing protocols, but rather from the core principle of testing patients on such a broad scale.

On the other hand, failure to provide the accurate diagnosis to these patients, however few they number, violates the principle of distributive justice. Patients with rare diseases are just as entitled to a diagnosis, with the ensuing community support, diagnostic closure, possibilities for treatment, and opportunities to engage in research as patients with common diseases, though this process if often lengthier and more challenging for rare diseases (Willmen et al. 2023). Resource limitations, like lack of access to genetic specialists, expensive or non‐conclusive testing, decreased awareness of rare diseases among clinicians, and more, hinder equitable distribution of accurate diagnoses to those with rare diseases (Rohani‐Montez et al. 2023). It is also important to note that funding and lobbying efforts by patient advocacy groups for research and resources toward rare diseases can unintentionally create disparities, as wealthier and better‐connected social groups may influence which diseases receive attention (Halley et al. 2023; Molina et al. 2015). Considering all these factors, the integration of rare diseases into diagnostic guidelines is desirable in order to accurately diagnose patients with rare diseases, which is also a consideration of beneficence in favor of these patients. Yet, this desirability is at odds with the consideration of non‐maleficence in reducing false positive results and in demanding disproportionate economic and scholastic resources for further research and development of testing and therapeutics (Gittus et al. 2023). The utility of receiving a diagnosis to the individual is also an important consideration, especially if the diagnoses lead to alterations in family planning (e.g., in vitro *fertilization*), actionable and effective therapy, or potential enrollment in clinical trials and experimental treatments. In cases in which there is no such available therapy, the utility of the diagnosis may depend on the desires of the particular patient. These benefits are dependent upon proper implementation of testing protocols for the appropriate patients.

The considerations above underscore the critical importance of promoting accessibility and clinician awareness of genetic testing. While this paper focuses primarily on cases in which considerations for and against testing are arguably equivocal, countless patients with clear clinical indication for genetic testing remain untested. For this group of patients, the burden of a prolonged diagnostic odyssey is an immeasurable, unquantifiable harm. Moreover, robust identification of genetic conditions have revolutionized management for patients with these disorders, emphasizing the gravity of missed therapeutics due to misdiagnosis or undiagnosis. Examples of such disorders include, but are not limited to, inherited cardiomyopathies, hereditary cancer syndromes, and developmental disorders, for which timely genetic testing and subsequent intervention immensely impacts quality of life and often lifespan for millions of people (see Watkins et al. 2011; Lynch and de la Chapelle 2003; Parenti et al. 2020 for examples). These delays lead to several, ongoing, potentially indefinite harms to patients and their families including ineffective and invasive interventions, exclusion from clinical trials, prolonged anxiety, opportunity costs, and more (Hofmann 2023). Estimates across a variety of conditions strongly support that genetic testing is widely underutilized, even when indications are clearly met (see Pederson and Narod 2024; Grant et al. 2025 for examples). This underutilization intersects with underserved populations, further widening health disparities in genetic diagnostics (Bather et al. 2025; Miller et al. 2023). Across the board, underutilization of genetic testing despite clear consistency of the clinical picture and an underlying genetic etiology is a rampant source of avoidable harms in the healthcare system. This concern merits significant attention, especially with respect to public health interventions, against the background of concerns about testing in low pre‐test probability conditions as discussed primarily in this paper.

Clinical trials and experimental treatments for rare diseases add another layer of complexity to this matter: should the absence of a possible treatment push us away from testing for a rare disease? As for many aspects of testing, the desires of the patient—reflecting the principle of autonomy—are important for answering questions like these. While a patient's request for testing alone, in the absence of other factors (e.g., clinical picture), may not justifiably lead to testing (especially given the harm of false positives), a patient's informed, consensual, and autonomous desire for testing is a viable consideration in the decision to test for a rare disease. That is, if a patient demonstrates capacity to autonomously authorize testing and, in addition, desires rare disease testing when prior diagnostic work‐up (e.g., physical examination, history, family history, laboratory data) is not incompatible with a rare disease entity, genetic testing may be acceptable.

The factors for testing discussed in Section 2 and their relationship to the considerations discussed in this section are summarized in Table 1.

**TABLE 1:** Considerations for rare disease testing.

Factor for rare disease testing	Rationale	Ethical consideration(s)	Example
Presentation should be atypical for a broader diagnostic category	Reduce false positives in testing	Non‐maleficence, beneficence	A patient with psychosis outside typical age range for schizophrenia
Common organic causes should be ruled out before testing	Avoid overlooking other diagnoses	Non‐maleficence, justice	Thyroid, B_12_, etc., for suspected dementia
Presentation should be consistent with a specific, treatable disorder entity or group	Ensure utility of diagnosis, lower likelihood of misleading VUS	Non‐maleficence, beneficence, justice	Family history of urea cycle disorders presenting as hepatic encephalopathy
Patient/family must have decision‐making capacity for testing and provide informed consent	Respect a patient's right (not) to know	Autonomy, non‐maleficence	An informed patient/family with a consistent clinical picture requests testing
Invasiveness of the test should be proportional to the utility of diagnosis. Less invasive tests are preferred	Minimize possible harm to patients undergoing testing	Non‐maleficence	A relatively non‐invasive test (e.g., saliva, blood draw) is preferred over an invasive test (e.g., bone marrow, cerebral spinal fluid)

## 4 Discrepancies in Real‐World Practices

The foregoing considerations for rare disease testing are aimed at reducing the number and harm of false positives, which involves both searching for more common diagnostic alternatives and keeping in mind the utility of a possible diagnosis to the patient (e.g., treatability). However, there are real‐world practices that may generate tension with these ethical considerations, which we explore in this section.

Newborn screening in eleven states in the United States of America include Krabbe disease, also known as globoid cell leukodystrophy, a lysosomal storage disease with a life expectancy of under two years in its infantile subtype (Matern et al. 2024; Orsini et al. 1993; Jain and De Jesus 2025). While newborn screening enables treatment with hematopoietic stem‐cell transplant, the effectiveness of screening and treatment have been criticized as being of dubious value, especially since many children are asymptomatic for years in non‐infantile subtypes (Ehmann and Lantos 2019). Considering the inability to robustly distinguish between subtypes of the disease through newborn screening, false positive rates, costly testing, and the encouragement of potentially risky, unnecessary intervention, current practices for newborn screening in Krabbe disease might be inconsistent with the ethical considerations detailed above (Lantos 2011).

The American College of Medical Genetics released a practice guideline suggesting genomic testing for Autism Spectrum Disorder (ASD), though the molecular diagnosis may not change the therapeutic approach or outlook for these patients (Manickam et al. 2021). In a survey of 461 adults with autism, almost half of participants responded that they thought genetic testing should not be done for ASD, with 83% expressing that it could increase discrimination toward autistic people (Byres et al. 2023). Moreover, genetic testing can inadvertently cause harm through breakdowns in risk communication (Rossi et al. 2013). While wide‐scale genetic testing can affect overarching approaches to and clinical research in ASD, the utility of genetic testing for current patients with ASD is crucial to balance, keeping in mind the potential risks for patients and families, including equivocal results or no corresponding change in treatment plan (Vorstman et al. 2017). Clarity in guidelines for implementation of testing can reasonably be expected to mitigate possible harms of inappropriate screening and testing discussed in the cases above.

Indeed, a majority of rare diseases lack guidelines to balance the potential benefits and harms of genetic testing. While patients with these diseases may stand much to gain from genetic testing, a lack of data and clarity maintains a corresponding lack of consensus as to how testing ought to be handled in a range of disorders, including neuromuscular disorders, ataxias, amyotrophic lateral sclerosis, psychiatric disorders, breast cancer, and more (Barp et al. 2021; Powell et al. 2010; Klepek et al. 2019; Koido et al. 2023; Pederson and Narod 2024). Legislative efforts, including the Genetic Information Nondiscrimination Act in the United States, reflect an increasing understanding of the possible harms of genetic data being exposed, but there are more efforts needed to protect vulnerable patients in the background of society at large (Feldman 2012; Suter 2018). However, the core of many of these ethical dilemmas is that much more data is required to understand the potential benefits and harms of screening or testing for a variety of disorders.

It is also important to emphasize that there are ethically‐significant differences between screening and testing for the purposes of this discussion. While they both rely on similar technologies, they have different purposes and thus different ethical considerations. Screening is often in a low pre‐test probability population and is intended primarily to identify individuals who may need further confirmatory evaluation for a condition that may benefit immensely from intervention. As a result, higher false positive rates compared to diagnostic testing are generally considered to be ethically tolerable, provided that further confirmatory testing is available to rule in or rule out disease (Hagenkord et al. 2020). By contrast, diagnostic testing is applied to patients for whom clinical suspicion is elevated for a particular disease. In this setting, there is a shift of risks and benefits because false positives generally have greater consequences, and accurate test results are needed to guide appropriate management (Hodge 2004). As a result, the considerations listed in Table 1 require further interpretation depending on whether screening or testing is being examined.

In short, there are important steps to take in evaluating apparent tension between theoretical desiderata for genetic testing and real‐world practices for a variety of diseases. Ultimately, these efforts aim to minimize harm to patients who may have rare diseases and maximize the benefits to the patients who do.

## 5 Implications and Challenges

Overall, it is critical to balance the individual‐level and population‐level benefits and harms of genetic testing, including not only the possibilities of accurate diagnoses and available therapeutic avenues, but also through the lens of false positives and utility of the diagnosis. As the field of medical genetics grows and diagnostic methods for ultra‐rare diseases outpace therapeutic avenues, careful consideration of the risks and benefits of testing ensures non‐maleficence toward the patient.

At the same time, widespread and accessible testing promotes distributive justice for those with undiagnosed conditions, who may benefit from a diagnosis regardless of whether treatment, or even a clinical trial, is available. It is also important to consider patient attributes associated with delayed diagnoses. For example, symptom onset before age of 30, female sex, having unmet psychological support, and more, all predict diagnostic delays (Faye et al. 2024). The promotion of testing for rare diseases through diagnostic guidelines may alleviate these disparities, but other limitations (e.g., access to a medical geneticist, interpretation of equivocal results, etc.) can pose additional hurdles on the road to diagnosis. Diagnosis can also be useful at a population‐level by increasing awareness among healthcare providers, advancing knowledge of rare diseases so that an accurate diagnosis may be established earlier. Increasing resources for rare diseases may lead to the development of new therapeutics, increasing and motivating the pool of research participants to advance treatment for future generations.

The challenge presented at the beginning of this article, in which rare diseases have deceptively common presentations, is a particularly salient and persistent dilemma in that it poses substantial difficulty even if the abovementioned recommendations are followed, owing to the extremely unique aspects of these cases. While there is no straightforward resolution to the ethical challenge of how and when to test for rare and ultra‐rare diseases, we have aimed to provide a basic set of principles to help guide decision‐making based on the utility of a diagnosis, the reduction harm to patients, and the aim of improving access to genetic testing. By carefully assessing the moral and practical challenges of diagnosing these rare diseases, we may start to pave the way to reducing the number of zebras left behind in a system designed for horses.

## Author Contributions

Rajeev Dutta and Changrui Xiao conceptualized the article. Rajeev Dutta drafted the article. Cathy Duong, Virginia Kimonis, and Changrui Xiao reviewed, improved, and modified the article.

## Funding

The authors have nothing to report.

## Ethics Statement

The authors have nothing to report.

## Conflicts of Interest

The authors declare no conflicts of interest.

## Data Availability

Data sharing not applicable to this article as no datasets were generated or analysed during the current study.
